# Analysis of the application effect of an automated eye position fixation training device in patients undergoing pterygium surgery: a two-year follow-up randomized controlled trial

**DOI:** 10.3389/fmed.2026.1764636

**Published:** 2026-06-02

**Authors:** Bei Chen, Meng Si, Fan Chen, Fei Jiang, Xueqin Chu, Huarong Wu, Rongfeng Liao

**Affiliations:** 1 Department of Ophthalmology, The First Affiliated Hospital of Anhui Medical University, Hefei, China; 2 Department of Ophthalmology, Anqing Municipal Hospital, Anqing, China; 3 Department of Pharmacy, Anqing Municipal Hospital, Anqing, China

**Keywords:** automated eye position fixation training device, conventional manual training, operative time, pterygium, recurrence rate

## Abstract

**Objective:**

This study aimed to evaluate the long-term effects of an automated eye position fixation training device (AEPFTD) on intraoperative cooperation, surgical efficiency, complications, and recurrence rates in patients undergoing pterygium surgery.

**Methods:**

In this single-center, double-blinded, prospective randomized controlled trial, 114 patients scheduled for unilateral primary pterygium surgery participated. Participants were randomly assigned to receive preoperative training using either the AEPFTD (experimental group, EG) or conventional manual training (control group, CG). We assessed primary outcomes of intraoperative cooperation in eye position, total operative time, and sustained maintenance of eye position. For secondary outcomes, we monitored recurrence rates at 6, 12, and 24 months after surgery, as well as specific intraoperative complications, including conjunctival suture loosening, corneal abrasion, and conjunctival tear. We analyzed outcomes with complete data using standard between-group statistical tests. For the recurrence, we also performed a per-protocol analysis and multiple imputation to handle missing follow-up data.

**Results:**

The EG had a significantly shorter total operative time than the CG (44.01 ± 4.96 min vs. 46.41 ± 4.73 min, *p* = 0.009). Adjustment counts for intraoperative eye position control showed no significant difference between groups (3 (1, 6) vs. 3 (1, 6) times, *p* = 0.734). Both groups showed comparable sustained eye-position maintenance (140.7 [105.3, 325.4] s vs. 144.1 [93.9, 281.8] s, *p* = 0.973). Both groups showed no significant differences in specific complications (conjunctival suture loosening, corneal abrasion, or conjunctival tear; all *p* > 0.05) or in the overall complication rate (10.53% vs. 17.54%, *p* = 0.236). In the PPS analysis, recurrence rates at 6 months, 1 year, and 2 years did not differ significantly. After we used MI to handle missing follow-up data, logistic regression also showed no significant difference in the 2-year recurrence rate (OR = 1.294, 95% CI: 0.295–5.683, *p* = 0.734).

**Conclusion:**

Compared with conventional manual training, the AEPFTD reduced operative time without significant differences in intraoperative cooperation, complications, or recurrence.

## 1 Introduction

Pterygium is a common fibrovascular growth of the ocular surface, especially in regions with high ultraviolet radiation (1, 2). It can cause redness, foreign-body sensation, blurred vision, and may result in complications such as corneal astigmatism and visual axis obstruction that impair vision and quality of life (3). Surgical excision is the main treatment, but postoperative recurrence is common, with reported rates ranging from 27–70% after simple excision and 5–14% with advanced techniques like conjunctival autografting (4–6). Recurrence leads to repeated surgeries and increased psychological and financial burden. Therefore, reducing recurrence and improving outcomes are key ophthalmic goals.

Researchers have improved outcomes through surgical refinements such as conjunctival autografting and amniotic membrane transplantation (5, 7) and by exploring adjuvant therapies, including mitomycin C, 5-fluorouracil, and VEGF inhibitors (8, 9). Despite these advances, recurrence persists. Recent evidence underscores the crucial role of patient cooperation in surgical success (10). Because pterygium excision is a delicate microsurgical procedure requiring strict gaze maintenance under local anesthesia, preoperative eye fixation training is critical to stabilize gaze, reduce anxiety, and aid surgical maneuverability, minimizing complications such as unintended eye movements, conjunctival tears, and suture disruption. The trials (11) show that manual training lowers uncommanded eye movements, operative time, recurrence, and suture loosening compared with no training, highlighting the value of behavioral interventions. However, manual training requires significant staff, varies in quality, and is difficult to standardize and scale. An automated, standardized training method is needed to address these issues.

Based on previous findings, this study introduces an automated eye-position fixation training device that uses visual targets and feedback sensors to guide patients through standardized eye exercises without requiring constant medical staff oversight. The device delivers consistent training, improves precision, and reduces nursing workload. This study assesses the device’s impact on intraoperative performance, surgical efficiency, complications, and long-term recurrence in pterygium patients. The two-year randomized controlled trial aims to develop a scalable, practical eye-position training tool for clinical care.

## 2 Materials and methods

### 2.1 Study design

This single-center, double-blinded (the surgeon and evaluator), parallel-group, prospective randomized controlled trial enrolled all participants. Researchers provided comprehensive information about the study’s objectives, procedures, risks, and benefits. All participants gave written informed consent. The study adhered to the ethical principles outlined in the Declaration of Helsinki. The Ethics Committee of Anqing Municipal Hospital approved the protocol (Ethical Approval No.: [2022] No. 81).

### 2.2 Study subjects

We evaluated consecutive patients diagnosed with unilateral primary pterygium at the Department of Ophthalmology, Anqing Municipal Hospital, between February 1 and November 30, 2023, and scheduled them for “Pterygium Excision Combined with Autologous Limbal Stem Cell Transplantation.” Among the 127 patients screened, we enrolled 114 who met the inclusion criteria and randomly assigned 57 to each group.

### 2.3 Inclusion and exclusion criteria

We enrolled patients who met all of the following inclusion criteria: (1) Were aged between 50 and 75 years; (2) had a clinical diagnosis of unilateral primary pterygium, with the pterygium head extending onto the cornea by more than 2 mm and requiring surgical intervention; (3) were scheduled to undergo “Pterygium Excision Combined with Autologous Limbal Stem Cell Transplantation”; we ensured that the same senior surgeon with over 10 years of experience performed all surgeries to minimize inter-surgeon variability; (4) were alert and oriented, with a Mini-Mental State Examination (MMSE) score ≥ 24, and possessed adequate Chinese literacy and comprehension skills to fully understand training instructions, study procedures, and cooperate with all assessments; (5) expressed willingness to participate voluntarily, provided written informed consent, and committed to completing the entire follow-up period; (6) had preoperative best-corrected visual acuity better than 0.4 (LogMAR) and astigmatism ≤ 3.0 D to exclude significant visual impairment that could interfere with cooperation during training and assessment; (7) owned a smartphone and could use basic smartphone functions for potential use in remote reminders and communication; (8) demonstrated good adherence by following training instructions accurately and maintaining compliance throughout the follow-up period.

We excluded patients who met any of the following criteria: (1) presented with uncontrolled severe systemic diseases, such as hypertension (systolic blood pressure ≥ 180 mmHg or diastolic blood pressure ≥ 110 mmHg), diabetes (fasting blood glucose ≥ 11.1 mmol/L or glycated hemoglobin ≥ 9.0%), or severe cardiac, pulmonary, hepatic, or renal insufficiency; (2) had local ocular conditions that could impair eye position control or cooperation, including prior ocular surgery or trauma, ocular motility disorders, severe dry eye syndrome, active ocular infection, keratitis, glaucoma, severe entropion or ectropion, or other conditions interfering with fixation training (e.g., nystagmus); (3) had craniofacial anatomical or functional abnormalities that may compromise intraoperative posture maintenance and cooperation, such as temporomandibular joint disorders or facial nerve paralysis; (4) had a history of diagnosed psychiatric or neurological disorders—including depression, anxiety disorders, epilepsy, Parkinson’s disease, or dementia—or currently used antipsychotic or antiepileptic medications; (5) had severe visual or hearing impairment that precluded comprehension of training instructions; (6) were judged by investigators to have poor adherence to follow-up, or investigators deemed it unlikely they would complete the two-year follow-up due to logistical factors (e.g., living at a distant location, lacking stable contact information, planning to relocate, or frequently traveling long-term) or refused to provide consent; (7) had a known allergy to commonly used intraoperative local anesthetic agents (e.g., lidocaine); (8) participated concurrently in other clinical trials that could potentially confound outcome assessment in this study.

### 2.4 Randomization and blinding

To ensure randomization and allocation concealment, an independent statistician who had no involvement in patient recruitment, intervention delivery, or outcome assessment generated a random sequence using SPSS software (version 26.0). Participants were randomly assigned in a 1:1 ratio to either the experimental group (EG) or the control group (CG). Allocation sequences were sealed in sequentially numbered, opaque, and tamper-evident envelopes. After written informed consent was obtained, a research nurse opened the envelopes sequentially to assign participants to their respective groups.

Due to the nature of the interventions, blinding of participants and the nurses administering the preoperative training was not feasible. However, to minimize potential bias, several key personnel were kept blinded to the group allocation throughout the trial. Specifically, the operating surgeon was not aware of the patient’s group assignment. This was achieved by ensuring the surgeon had no involvement in the preoperative training or the randomization process. Furthermore, the personnel involved in intraoperative data collection (e.g., circulating nurses recording operative times), the ophthalmologists conducting all postoperative follow-up assessments (evaluating recurrence and complications), and the statistician performing the final data analysis remained fully blinded to the group assignments. This strict separation of roles was maintained to ensure the objectivity of both intraoperative performance and long-term outcome assessment.

### 2.5 Sample size estimation

This study was designed as a superiority trial. To ensure an adequate sample size, an *a priori* power analysis was conducted. The target effect size was informed by a preliminary pilot study conducted on 20 patients scheduled for pterygium surgery (10 in the experimental group and 10 in the control group). In this pilot study, we evaluated total operative time as a primary outcome. The mean operative time was 43.61 ± 5.02 min for the experimental group and 47.26 ± 4.69 min for the control group. Based on these data, we calculated the effect size (Cohen’s d) to be approximately 0.75. To be conservative and ensure sufficient power, we selected a target effect size of d = 0.8, a conventional large effect size supported by our pilot data. Sample size calculation was performed using G*Power software (version 3.1.9.7) for an independent-samples *t*-test. With a two-tailed *α* error probability set at 0.05 and a desired power (1-*β*) of 80%, the calculation indicated that a minimum of 26 participants per group was required. To account for a potential dropout rate of 10%, the target sample size was inflated to 29 participants per group, yielding a minimum total sample size of 58.

Pterygium is a common ophthalmic condition, and patient recruitment was feasible within the planned study period. To enhance the statistical power of our study and increase the robustness and reliability of our findings, especially for secondary outcomes such as recurrence and complications, we decided *a priori* to continue recruitment beyond the minimum required number. Ultimately, 114 patients were enrolled during the study’s recruitment phase.

### 2.6 Design and principle of the AEPFTD

The AEPFTD used in this study was developed to provide a standardized, automated, and reproducible method for preoperative eye position fixation training, addressing the limitations of conventional manual instruction. The device is designed to be user-friendly for both patients and nursing staff and is easily implementable in a standard clinical setting.

The core components of the device relevant to its clinical function include:

*A visual guidance system*: This consists of an adjustable stand with an array of LED lights. The lights serve as fixation targets, simulating the directional gaze commands required during surgery (e.g., “look right,” “look down”). The light intensity is adjustable to ensure patient comfort and visibility.

*An auditory prompt system*: A built-in audio module delivers clear, pre-recorded verbal commands that are synchronized with the corresponding visual cues (e.g., the command “look right” is paired with the illumination of the right-side LED). This audiovisual synchronization helps reinforce patient learning.

*A programmable control unit*: The device operates on a pre-programmed, progressive training algorithm. The software guides the patient through a structured session, starting with shorter fixation periods (e.g., 30 s per direction) and gradually increasing the duration up to 180 s. This stepwise progression allows for adaptive learning and endurance building. An automated countdown provides continuous feedback to the patient, encouraging sustained fixation.

**Figure 1:**
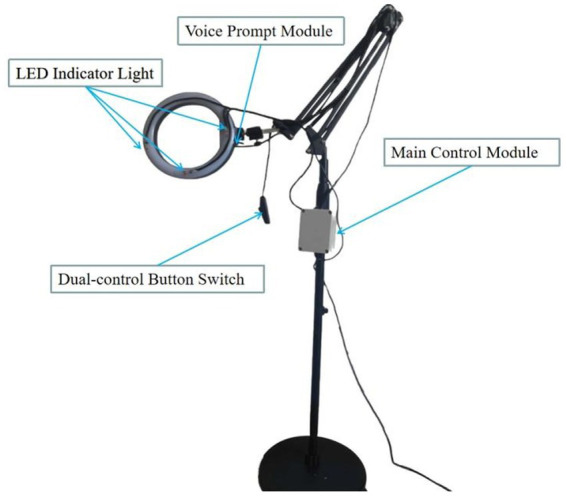
Automated eye position fixation training device.

The entire device is lightweight (approx. 3.5 kg), portable, and powered via a standard USB interface, enabling flexible use across various clinical environments, such as outpatient clinics and inpatient wards. Its design prioritizes ease of use, requiring minimal setup by a nurse, after which the patient can complete the training session autonomously. This automated nature ensures that every patient receives a training protocol of consistent quality, intensity, and duration, thereby guaranteeing the reproducibility of the intervention (Figure 1).

### 2.7 Intervention measures

All enrolled patients underwent “Pterygium Excision Combined with Autologous Limbal Stem Cell Transplantation” under local anesthesia. Under local anesthesia, patients remained conscious and retained voluntary eye movements; therefore, they were still required to cooperate with the surgeon’s instructions and maintain a stable gaze in specific directions during the procedure. The purpose of the device was not to mechanically immobilize the eye, but to provide standardized preoperative training to improve command comprehension, gaze stability, and intraoperative cooperation.

**Figure 2:**
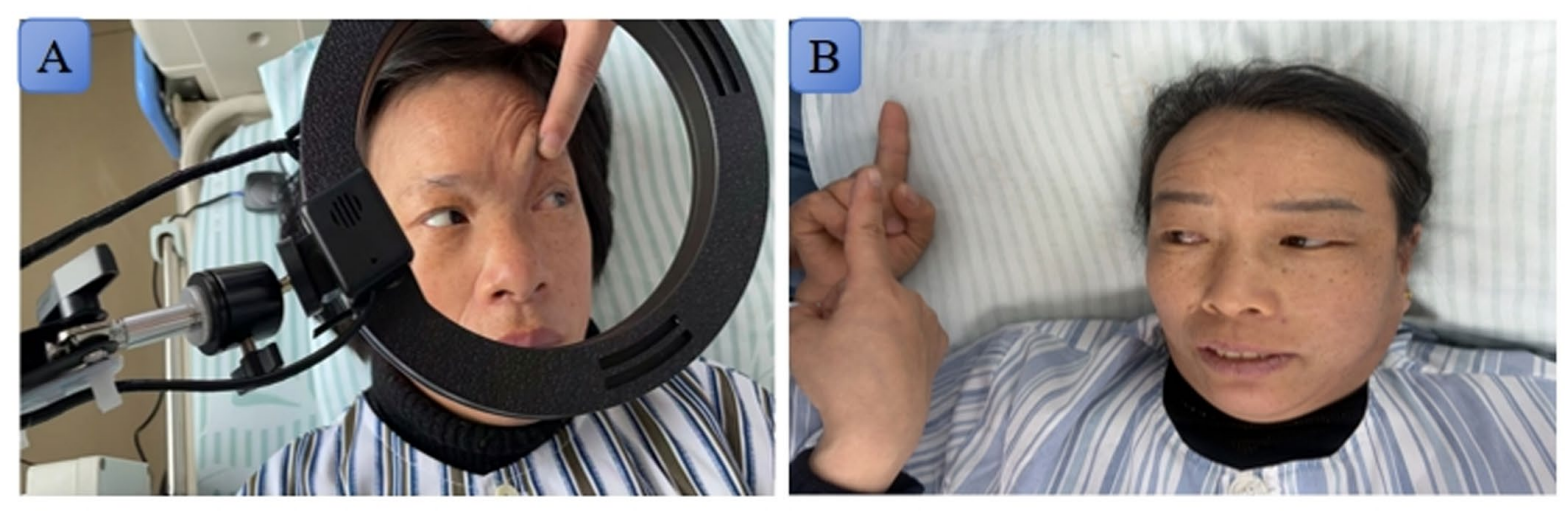
Examples of preoperative eye-position fixation training in the two patient groups. **(A)** Automated eye position fixation training; **(B)** Conventional manual training.

The procedures were performed by the same team of senior ophthalmologists to ensure standardization of surgical techniques. The two groups received different preoperative eye position fixation training regimens. The experimental group (EG) underwent training using the AEPFTD, while the control group (CG) received conventional manual eye position training (as shown in Figure 2).

*Control group (conventional manual training)*: Patients in the control group (CG) received training administered by three uniformly trained ward nurses. The sessions were conducted in a quiet ward environment with patients seated. A nurse used the right index finger as a visual target, positioned approximately 33 cm in front of the patient’s operative eye. Verbal commands (“look right,” “look down,” “look left”) were delivered in a fixed sequence while the nurse moved the finger to guide the patient’s eye movements and maintain fixation. Training commenced 2 days before surgery and was conducted twice daily (morning and afternoon), with each session lasting 30 min.

*Experimental group (automated device training)*: Patients in the experimental group (EG) were trained using the self-developed AEPFTD. Prior to the first session, a nurse provided a brief explanation of the device’s auditory and visual commands. During training, patients were positioned in a supine posture. A nurse adjusted the device to position the light source 30 cm from the operative eye and confirmed patient understanding of the procedure. The session then proceeded automatically according to the device’s preset program. The device guided eye movements and maintained fixation through synchronized voice commands and directional LED cues. Training followed a stepwise progressive algorithm, gradually increasing the duration of sustained gaze fixation in each direction from 30 s to 180 s, forming a complete 30-min session. The training schedule and frequency (twice daily for 2 days preoperatively) were identical to those of the CG. This protocol ensured comparability between the two groups across all variables except for the mode of training.

### 2.8 Observation indicators

This study employed a multidimensional set of outcome measures. All data were collected by uniformly trained research personnel who were blinded to group assignment, ensuring consistent and objective assessment.

Primary outcome indicators focused on intraoperative performance:

(1) *Intraoperative eye position cooperation*: Two circulating nurses, who were blinded to the patient’s group allocation, were responsible for data collection. They were present in the operating room and simultaneously observed the live video feed from the surgical microscope’s camera on a monitor. Both nurses were extensively trained on a detailed Standardized Operational Protocol (SOP) prior to the trial, which provided explicit, operationalized definitions for each event to be counted:

- *Intraoperative eye position adjustment count*: Defined as the total number of explicit verbal commands (e.g., “look right a bit more”) or manual interventions (e.g., using forceps to gently guide the globe) made by the surgeon in response to a patient’s eye position deviating ≥5° from the target gaze direction. The 5° threshold was visually estimated by the trained observers using the markings on the surgical field as a reference.
- *Voluntary eye movement count*: Defined as the total number of spontaneous, uncommanded eye movements (including saccades, drifts, or rolls) that were of sufficient magnitude to either interrupt the surgical maneuver, require the surgeon to pause, or pose a risk to ocular structures. Minor, insignificant nystagmoid movements were not counted.
- *Active eye position adjustment count*: Defined as the total number of times the patient, without receiving a direct command, proactively and correctly adjusted their gaze back to the required target position after a minor deviation. This indicates a high level of patient engagement and understanding of the surgical requirements.
- *Command direction compliance error count*: Defined as the total number of instances where the patient’s eye movement failed to match the surgeon’s explicit verbal command in direction or execution. This includes moving in the wrong direction (e.g., the surgeon says “look up,” but the patient looks down), not moving at all, or making an incorrect type of movement (e.g., rolling the eye rather than holding a fixed gaze).

(2) *Surgical efficiency*: Measured by total operative time, defined as the duration from the first incision of the bulbar conjunctiva to the tying of the final suture, recorded using a stopwatch.
(3) *Eye position maintenance ability*: This was assessed by measuring the maximum single gaze maintenance time. To ensure accuracy and feasibility, this metric was determined retrospectively from the surgical video recordings. This retrospective approach was chosen because the dynamic and demanding nature of the intraoperative environment makes it challenging to perform accurate manual timing concurrently with other clinical duties. Post-hoc video analysis allows for focused, uninterrupted, and verifiable measurement. The measurement was performed by two of the study’s authors (Meng Si and Xueqing Chu). Crucially, both authors were blinded to the patients’ group allocation throughout the entire data collection and analysis process. After the surgery, the two authors viewed the high-definition video for each patient together to identify potential periods of sustained fixation. For each identified period, they then independently timed the duration using handheld digital stopwatches (with a precision of 0.1 s). The fixation period was timed from the video frame in which the surgeon confirmed the eye was correctly positioned and stable, and ended at the frame at which the first significant eye movement (e.g., saccade, drift, or wobble) breaking fixation was observed. After reviewing the entire video, the longest duration recorded for each patient was identified as the patient’s maximum single-gaze maintenance time. The final value used for analysis was the average of the two authors’ times for that specific longest-duration event, to minimize measurement error.

Secondary outcome indicators focused on long-term efficacy and safety:

(1) *Recurrence rate*: Evaluated at 6 months, 1 year, and 2 years postoperatively by a senior physician blinded to group allocation using slit-lamp examination. Recurrence was defined as neovascularization with proliferative tissue extending more than 1 mm onto the cornea from the limbus.
(2) *Intraoperative complications*: The occurrence of conjunctival suture loosening, corneal abrasion (defined as a linear epithelial defect confirmed microscopically by the surgeon), and conjunctival tear was documented during surgery.

To minimize observer bias and ensure data accuracy, a real-time data verification process was implemented. The two nurses independently but simultaneously recorded the counts on separate standardized forms. Throughout the procedure, they were permitted to have brief, quiet discussions to confirm an event’s occurrence, especially for ambiguous situations, thereby reaching an immediate consensus. If a consensus could not be reached through this brief discussion, the chief operating surgeon was asked to make a final verbal adjudication on the spot, based on his immediate assessment of the event in question (while remaining blind to the patient’s group allocation). This structured, real-time process was designed to standardize data collection and resolve potential observer disagreements as they occurred.

### 2.9 Statistical analysis

Statistical analysis was performed using SPSS software (version 26.0). Continuous variables were assessed for normal distribution using the Shapiro–Wilk (S-W) test. Normally distributed continuous variables were expressed as mean ± standard deviation (Mean ± SD) and compared between groups using the independent samples t-test. Non-normally distributed data were presented as medians with interquartile ranges [M (IQR)] and analyzed using the Mann–Whitney U test. Categorical variables were presented as frequencies (percentages), and group comparisons for complete data were conducted using the Chi-square test or Fisher’s exact test.

During the 2-year follow-up, 8 patients were lost to follow-up (an overall attrition rate of 7.0%). Because loss to follow-up affected recurrence outcomes, 106 patients who completed the entire follow-up were included in the Per-Protocol Set (PPS) analysis. To minimize potential bias from missing follow-up data, multiple imputation (MI) with 5 imputations was performed for the recurrence analysis. After imputation, a univariate binary logistic regression model was used to compare the 2-year recurrence rate between groups and to estimate the pooled odds ratio (OR), 95% confidence interval (CI), and *p*-value. All statistical tests were two-sided, and *p*-values < 0.05 were considered statistically significant.

## 3 Results

### 3.1 Comparison of baseline characteristics between the two groups

**Figure 3:**
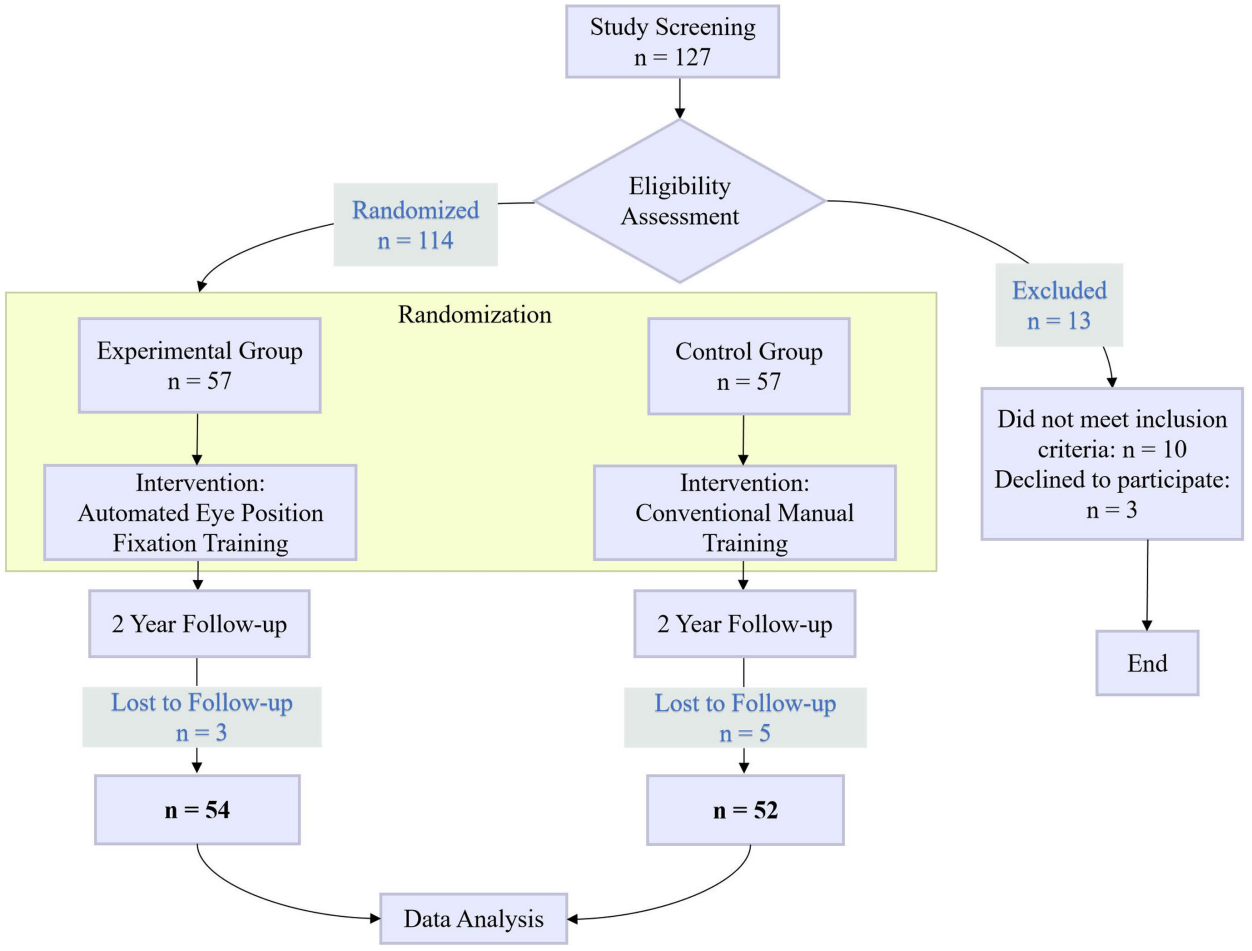
Study flow diagram.

A total of 127 patients were screened for eligibility. Of these, 114 met the inclusion criteria and were randomized: 57 to the experimental group (EG) and 57 to the control group (CG). During the 2-year follow-up period, three patients (5.3%) in the EG and five patients (8.8%) in the CG were lost to follow-up, all after the 1-year visit. Reasons for loss to follow-up included inability to contact (*n* = 7) and death (*n* = 1, in the CG). Therefore, 106 patients (54 in the EG and 52 in the CG) completed the entire follow-up and were included in the PPS recurrence analysis. In addition, all 114 enrolled patients were included in the recurrence analysis after multiple imputation of missing follow-up data. The participant flow is detailed in Figure 3.

As shown in Table 1, there were no statistically significant differences between the two groups with respect to age, gender, operative eye, pterygium grade, distance of corneal invasion, preoperative best-corrected visual acuity, astigmatism, or MMSE score (all *p* > 0.05), indicating well-balanced baseline characteristics and adequate intergroup comparability.

**Table 1:** Comparison of baseline characteristics between the two groups [*M*(IQR)/*n* (%)].

Characteristic	EG (*n* = 57)	CG (*n* = 57)	Statistic	*p*
Age (years)	64 (50,75)	64 (50,75)	Z = 0.000	1.000
Gender			χ^2^ = 0.022	0.882
Male	32 (56.1%)	29 (50.9%)		
Female	25 (43.9%)	28 (49.1%)		
Operative eye			χ^2^ = 0.019	0.889
Right eye	30 (52.6%)	28 (49.1%)		
Left eye	27 (47.4%)	29 (50.9%)		
Pterygium grade			χ^2^ = 2.574	0.276
T1 (2–4 mm)	45 (78.9%)	42 (73.7%)		
T2 (4–6 mm)	11 (19.3%)	15 (26.3%)		
T3 (>6 mm)	1 (1.8%)	0 (0.0%)		
Distance of pterygium invasion into cornea (mm)	3 (2,7)	3 (2,5)	Z = −0.436	0.663
Preoperative best-corrected visual acuity	0.4 (0.1,0.4)	0.4 (0.1,0.4)	Z = −0.074	0.941
Degree of astigmatism (D)	2.26 (1.45,2.88)	2.18 (1.46,2.89)	Z = −0.082	0.935
MMSE score (points)	27 (24,32)	27 (24,32)	Z = −0.264	0.792
Smoking	18 (31.6)	20 (35.1)	χ^2^ = 1.878	0.171
Alcohol consumption	21 (36.8%)	23 (40.4%)	χ^2^ = 0.668	0.414
Hypertension	25 (43.9)	23 (40.4)	χ^2^ = 0.242	0.623
Diabetes	12 (21.1)	10 (17.5)	χ^2^ = 0.574	0.449

### 3.2 Comparison of intraoperative eye position cooperation

As shown in Table 2, no significant differences were observed between the EG and the CG in any of the indicators of eye position cooperation (all *p* > 0.05). Specifically, the two groups exhibited comparable performance with respect to intraoperative eye position adjustment count [3 (1, 6) vs. 3 (1, 6), *p* = 0.734], voluntary eye movement count [1 (0, 3) vs. 1 (0, 4), *p* = 0.422], active eye position adjustment count [0 (0, 2) vs. 0 (0, 2), *p* = 0.319], and command direction compliance error count [0 (0, 4) vs. 0 (0, 3), *p* = 0.420] (Table 2).

**Table 2:** Comparison of intraoperative eye position cooperation between the two groups [*M*(IQR)].

Eye position cooperation	EG (*n* = 57)	CG (*n* = 57)	*Z*	*p*
Intraoperative eye position adjustment count (times)	3 (1,6)	3 (1,6)	−0.340	0.734
Voluntary eye movement count (times)	1 (0,3)	1 (0,4)	−0.803	0.422
Active eye position adjustment count (times)	0 (0,2)	0 (0,2)	−0.997	0.319
Command direction compliance error count (times)	0 (0,4)	0 (0,3)	−0.807	0.420

### 3.3 Comparison of surgical efficiency and eye position maintenance ability

As shown in Table 3, the total operative time was significantly shorter in the EG than in the CG (44.01 ± 4.96 min vs. 46.41 ± 4.73 min, *t* = 2.641, *p* = 0.009). With regard to eye position maintenance ability, there was no significant difference in the median intraoperative maximum single gaze maintenance time between the two groups [140.7 (105.3, 325.4) s vs. 144.1 (93.9, 281.8) s; Z = −0.034, *p* = 0.973].

**Table 3:** Comparison of surgical efficiency and eye position maintenance ability between the two groups [*M*(IQR)/Mean ± SD].

Indicators	EG (*n* = 57)	CG (*n* = 57)	Statistic	*p*
Total operative time (minutes)	44.01 ± 4.96	46.41 ± 4.73	t = 2.641	0.009
Intraoperative maximum single gaze maintenance time (seconds)	140.7 (105.3,325.4)	144.1 (93.9,281.8)	Z = −0.034	0.973

### 3.4 Comparison of complication occurrence

As shown in Table 4, the overall complication rate was lower in the EG (10.53% [6/57]) than in the CG (17.54% [10/57]), but this difference was not statistically significant (χ^2^ = 1.163, *p* = 0.281). The incidence rates of specific complications were also comparable between the two groups: conjunctival suture loosening (5.26% [3/57] vs. 8.77% [5/57]), corneal abrasion (3.51% [2/57] vs. 5.26% [3/57]), and conjunctival tear (1.75% [1/57] vs. 3.51% [2/57]) (all *p* > 0.05 for intergroup comparisons).

**Table 4:** Comparison of complication occurrence between the two groups [*n* (%)].

Complication type	EG (*n* = 57)	CG (*n* = 57)	Statistic	*p*
Conjunctival suture loosening	3 (5.26)	5 (8.77)	Fisher’s	0.716
Corneal abrasion	2 (3.51)	3 (5.26)	Fisher’s	1.000
Conjunctival tear	1 (1.75)	2 (3.51)	Fisher’s	1.000
Total complications	6 (10.53)	10 (17.54)	χ^2^ = 1.163	0.281

### 3.5 Comparison of postoperative recurrence rates

In the PPS analysis, recurrence rates in the EG (*n* = 54) and the CG (*n* = 52) were as follows: at 6 months postoperatively, 1.85% (1/54) vs. 1.92% (1/52), *p* = 1.000; at 1 year, 1.85% (1/54) vs. 5.77% (3/52), *p* = 0.618; and at 2 years, 5.56% (3/54) vs. 7.69% (4/52), *p* = 1.000. No statistically significant differences were observed at any time point in the PPS analysis (Table 5).

**Table 5:** Comparison of postoperative recurrence rates between the two groups [*n* (%)].

Time point	EG (*n* = 54)	CG (*n* = 52)	Statistic	*p*
6 months postoperatively	1 (1.85)	1 (1.92)	Fisher’s	1.000
1 year postoperatively	1 (1.85)	3 (5.77)	Fisher’s	0.358
2 years postoperatively	3 (5.56)	4 (7.69)	Fisher’s	0.713

During the 2-year follow-up period, 8 patients were lost to follow-up. Missing data were handled using multiple imputation (with 5 imputations generated). Subsequently, a binary logistic regression analysis was performed with 2-year postoperative recurrence as the dependent variable and group allocation as the independent variable. The results showed no statistically significant difference in the 2-year recurrence rate between the experimental and control groups (OR = 1.294, 95% CI: 0.295–5.683, *p* = 0.734).

## 4 Discussion

This single-center, two-year randomized controlled trial evaluated the application of an AEPFTD in patients undergoing pterygium surgery. The results demonstrated comparable intraoperative eye position cooperation between the EG and the CG, confirming the efficacy of the standardized automated regimen. Although the absolute reduction in total operative time was modest (approximately 2.4 min), this finding likely reflects a smoother surgical workflow with fewer intraoperative interruptions, such as unplanned eye movements or repeated gaze adjustments, rather than representing a clinically dominant time-saving effect. No significant difference in complication rates was observed, supporting the safety of the automated approach. In both the MI and PPS analyses, the two-year recurrence rate showed no statistically significant difference, confirming that the device provides comparable long-term efficacy. The AEPFTD can reduce operative time without compromising intraoperative cooperation or long-term recurrence rates, making it an effective alternative for preoperative preparation. Although this study focused on pterygium excision, the core AEPFTD mechanism—training sustained gaze and directional eye movements—is highly transferable. It could be applied to other ophthalmic procedures under topical anesthesia that require patient cooperation, such as cataract surgery, intravitreal injections, and refractive surgeries.

The current study found that the AEPFTD did not demonstrate superiority over traditional manual training with respect to intraoperative eye position cooperation or maintenance ability (all *p* > 0.05). This finding aligns with the broader consensus in previous reports (12, 13), which highlight that structured, standardized simulated training regimens are highly effective in optimizing ophthalmic surgical outcomes. Our results build upon this evidence by demonstrating that an automated delivery system can achieve clinical outcomes fully equivalent to those of high-quality, individualized manual instruction.

The clinical significance of the 2.4-min reduction in operative time should be interpreted in context. While this difference may appear modest in absolute terms, several factors suggest it has practical relevance: (1) in microsurgical procedures requiring sustained concentration, even small reductions in operative duration may translate into reduced surgeon fatigue and improved precision, particularly in high-volume surgical settings. (2), the time reduction likely reflects fewer intraoperative disruptions due to better patient cooperation, which may indirectly contribute to improved surgical quality and reduced complication risk, although our study did not show a statistically significant difference in complications (10.53% vs. 17.54%, *p* = 0.236). (3), from a health systems perspective, small per-case time savings can accumulate to meaningful efficiency gains when scaled across multiple procedures.

However, we acknowledge that the clinical importance of this finding should not be overstated. The magnitude of the time difference is relatively small, and whether it translates into meaningful patient-centered benefits—such as reduced anxiety, improved comfort, or better long-term outcomes—remains uncertain and requires validation in larger studies. Therefore, we present this as a secondary advantage of the automated training device, rather than its primary justification.

Evaluating the real-world applicability of the AEPFTD involves assessing its cost-effectiveness, training requirements, and comparing it with simpler alternatives. Although the device has an initial hardware cost, it offers significant long-term value by being highly efficient with human resources. Unlike labor-intensive manual training, which requires extensive one-on-one nursing time, the AEPFTD only needs a few minutes for setup, after which the patient trains autonomously. This drastically reduces the nursing workload. In terms of training, the device is highly intuitive; our experience shows that patients typically master its use after a single, brief demonstration (<5 min). Compared to simpler alternatives such as intraoperative verbal coaching, any form of preoperative preparation is superior because it is proactive. The AEPFTD’s key advantage over manual training lies in its high standardization and reproducibility, which eliminate inter-operator variability and ensure consistent quality of care. Therefore, the device represents a scalable and efficient solution for standardizing preoperative patient training.

Previous literature suggests that simulated preoperative training can significantly improve eye position cooperation. For instance, in refractive surgery, such training has been associated with longer, more stable target-gaze duration and reduced saccadic frequency during procedures (12). Similarly, simulated eye-position training for pterygium surgery has been shown to decrease intraoperative deviations and directional errors, as well as shorten operative time, contributing to smoother surgical execution (13). These findings suggest that standardized audiomotor training helps patients pre-establish cognitive and motor patterns for required eye positions, thereby enhancing intraoperative compliance.

The results of this study revealed that, although the overall complication rate was numerically lower in the AEPFTD-trained group (10.53% vs. 17.54%), this difference did not reach statistical significance. Similarly, the incidence rates of specific complications—conjunctival suture loosening, corneal abrasion, and conjunctival tear—showed no significant differences between the two groups. This may indicate that both training modalities achieved comparable effects in enhancing intraoperative eye position cooperation and maintenance, thereby contributing similarly to complication prevention. Supporting this interpretation, a previous study (14) reported that patients who underwent preoperative eye and head position training required significantly fewer intraoperative adjustments than untrained patients. In that study, the untrained group experienced 11 complications (including suture loosening, conjunctival tear, corneal abrasion, and dry eye), compared to only 3 in the trained group, indicating a significantly lower complication rate with training. Another study (13) confirmed that preoperative simulated eye-position training was associated with significantly reduced counts of intraoperative eye deviation, eye movement errors, and spontaneous eye movements, as well as a lower incidence of postoperative complications, compared to no training. Collectively, these findings highlight a core mechanism by which standardized eye-position training may reduce complication risk: improved patient comprehension of commands, enhanced gaze stability, and reduced disorganized eye movements.

Regarding long-term outcomes, the MI analysis in this study showed no statistically significant difference in pterygium recurrence rate at 2 years postoperatively between the Automated Device group and the manual training group (*p* = 0.734). Consistent with the PPS analysis (which included only protocol-compliant patients), this indicates that the automated training provides equivalent long-term efficacy compared to conventional methods. Although a numerical reduction in recurrence was observed in the experimental group, this difference did not reach statistical significance, which warrants further discussion.

A plausible explanation is that good intraoperative eye position cooperation is fundamental to surgical success, as it directly affects the precision of surgical maneuvers and minimizes tissue invasiveness. Known risk factors for pterygium recurrence after surgery include recurrent lesions, pterygium size > 6.7 mm, fleshy (T3 grade) severity, a preoperative neutrophil-to-lymphocyte ratio ≥ 2.01, and the potentially debatable factor of younger age (< 45 years) (15, 16). However, beyond these risk factors, the surgical procedure itself is a key determinant of recurrence. Studies indicate that incomplete excision of pterygium lesion tissue is a core cause of recurrence. If the fibrovascular stroma of the pterygium or residual conjunctival epithelial cells are not completely removed during surgery, the remaining tissue may continue to proliferate (1, 17). Preoperative systematic eye position training can improve patient cooperation during surgery. For example, trained patients exhibit significantly fewer intraoperative adjustments in eye and head position than inadequately trained patients (18, 19). More stable intraoperative eye position maintenance provides better and more consistent exposure of the surgical field. This enables surgeons to excise the lesion more thoroughly and perform suturing during limbal stem cell transplantation with greater precision. These procedural advantages are critical for reducing the risk of postoperative recurrence. In this study, the automated training device, through its standardized, programmable protocol, likely ensured that all patients received eye-position fixation training with consistent intensity, duration, and quality standards. This reduced the variability and potential communication bias inherent in manual instruction. The high level of training consistency may have contributed to more uniform and reliable intraoperative cooperation in eye position.

Why did the numerical reduction in the 2-year recurrence rate not reach statistical significance? This is likely due to the relatively small sample size and the overall low incidence of recurrence. From a statistical perspective, the 6-month recurrence rate was extremely low in both groups (only 1.75%). Even with follow-up extended to 2 years, the absolute number of recurrence events remained small. Given the rarity of these events, the current study may lack sufficient statistical power to detect a meaningful intergroup difference in recurrence. Pterygium recurrence is a chronic, multifactorial process. Early postoperative inflammatory reactions initiate recurrence, while a long-term, unstable ocular surface environment may promote its progression (20, 21). From a clinical and biological perspective, early recurrences (within 6 months) are usually driven by intense, acute postoperative inflammation or individual wound-healing patterns. These factors can mask the subtle benefits of surgical precision. In contrast, late recurrences (1 to 2 years) occur more slowly and are often due to the gradual growth of tiny fibrovascular remnants left behind during surgery. The AEPFTD offered more sustained ocular stability, likely allowing a more meticulous and thorough microscopic excision. This complete clearance eliminates the “seeds” of long-term efficacy, which takes longer to show clinically.

Innovations and Limitations of This Study: The study’s innovations include the following. First, we designed and applied a standardized, programmable AEPFTD specifically for ophthalmic surgery. This device ensured objective consistency in training, reduced human intervention and inter-operator variability, and saved human resources. While conventional manual training requires 120 min of dedicated one-on-one nursing supervision per patient (30 min per session, 4 sessions in total), the AEPFTD requires only a brief setup of a few minutes per session, thereby freeing nurses to attend to other clinical duties and optimizing human resource allocation. Second, this study used a rigorous randomized controlled trial design to provide long-term follow-up evidence comparing this device with conventional training in a real-world clinical setting. The findings regarding its impact on surgical efficiency and long-term recurrence risk add to the evidence base for perioperative behavioral interventions.

However, this study has several limitations: (1) As a single-center study with relatively narrow inclusion criteria (e.g., requiring smartphone ownership and adequate literacy), the generalisability of the findings needs to be validated in diverse populations and multicentre settings, particularly among older or less technologically adept patients. (2) Blinding and Potential for Performance Bias: A significant limitation of this trial is that the nature of the interventions made it impossible to blind the participants and training nurses. This lack of blinding introduces a potential for performance bias. For example, patients in the experimental group, aware they were using a novel automated device, might have been more motivated or engaged (a Hawthorne effect). Conversely, nurses in the control group might have inadvertently provided more encouragement to “compete” with the device. While we attempted to mitigate this by blinding the outcome assessors (surgeons, postoperative evaluators, and statisticians), this potential bias in behavioral outcomes, such as intraoperative cooperation, cannot be entirely ruled out and should be considered when interpreting the results. (3) Although the sample size was calculated *a priori*, the statistical power for outcomes with low incidence rates (e.g., specific complications) may still be insufficient. (4) Despite appropriate statistical handling (e.g., multiple imputation), loss to follow-up may have introduced potential bias in the long-term outcome assessment. (5) The automated device was a self-developed prototype; its standardized production, scalability, and long-term technical stability require further evaluation. (6) The training protocol used a fixed mode and did not allow for individualized adjustments. Additionally, its clinical efficacy has been validated only in pterygium surgery, meaning specific training software protocols may need to be customized and tested before being applied to other ocular surgeries. (7) Some intraoperative observation indicators, despite rater training, still involved a degree of subjective judgment. In light of these limitations, future research should employ large-sample, multicentre designs and incorporate more objective assessment tools, such as eye-tracking technology.

## Data Availability

The raw data supporting the conclusions of this article will be made available by the authors, without undue reservation.
